# Evaluating housing quality, health and safety using an Internet-based data collection and response system: a cross-sectional study

**DOI:** 10.1186/1476-069X-9-69

**Published:** 2010-11-12

**Authors:** Mari Turunen, Ari Paanala, Juha Villman, Aino Nevalainen, Ulla Haverinen-Shaughnessy

**Affiliations:** 1 National Institute for Health and Welfare, Department of Environmental Health, P.O. Box 95, FIN-70701 Kuopio, Finland

## Abstract

**Background:**

Typically housing and health surveys are not integrated together and therefore are not representative of population health or national housing stocks. In addition, the existing channels for distributing information about housing and health issues to the general public are limited. The aim of this study was to develop a data collection and response system that would allow us to assess the Finnish housing stock from the points of view of quality, health and safety, and also to provide a tool to distribute information about important housing health and safety issues.

**Methods:**

The data collection and response system was tested with a sample of 3000 adults (one per household), who were randomly selected from the Finnish Population Register Centre. Spatial information about the exact location of the residences (i.e. coordinates) was included in the database inquiry. People could participate either by completing and returning a paper questionnaire or by completing the same questionnaire via the Internet. The respondents did not receive any compensation for their time in completing the questionnaire.

**Results:**

This article describes the data collection and response system and presents the main results of the population-based testing of the system. A total of 1312 people (response rate 44%) answered the questionnaire, though only 80 answered via the Internet. A third of the respondents had indicated they wanted feedback. Albeit a majority (>90%) of the respondents reported being satisfied or quite satisfied with their residence, there were a number of prevalent housing issues identified that can be related to health and safety.

**Conclusions:**

The collected database can be used to evaluate the quality of the housing stock in terms of occupant health and safety, and to model its association with occupant health and well-being. However, it must be noted that all the health outcomes gathered in this study are self-reported. A follow-up study is needed to evaluate whether the occupants acted on the feedback they received. Relying solely on an Internet-based questionnaire for collecting data would not appear to provide an adequate response rate for random population-based surveys at this point in time.

## Background

The characteristics of housing hold both social and economic importance for people, but more detailed information on how the quality of housing is linked to health and safety is limited [1,2]. Data collection methods are critical to the efficiency of population studies. Questionnaire-based study methods have been used in previous studies investigating occupant health and safety [3-7]. In today's communication society, web-based methods appear to be a natural development of methodology. Web-based questionnaires, usually in tandem with a paper questionnaire, have been used in several health-related studies [8,9] with satisfactory response rates. Comparisons between responses to paper and Internet questionnaires [10] have shown them to yield similar results. However, in one Norwegian study [11] there was no meaningful increase in the response rate as a result of an option to respond via the Internet.

In Finland in 2007, 75% of persons aged 15-74 used the Internet at least once a week [12], which means that a majority of people have access to the Internet. Thus we were encouraged to develop the option for an Internet response to the questionnaire along with the traditional paper form.

### Aims of the study

The overall aim of the study was to develop a data collection and response system that would provide a means to assess the Finnish housing stock from the points of view of quality, health and safety. An option for respondents to reply via the Internet was included in the system. The system was also intended to ascertain people's information needs concerning housing and health, to explore spatial information in relation to occupant health issues, and to provide a tool for distributing information about important housing health and safety issues. The latter was accomplished by offering the respondents the possibility to receive individualised feedback based on their responses, which, together with an option to respond via the Internet, was expected to enhance the response rate.

In this article we describe the data collection and response system and present the main results of the population-based testing of the system.

## Methods

### Questionnaire development

Various sources contributed to the design of the questionnaire [13-15]. The questionnaire was tested among the Institute's employees, while a small city-based pilot study was also carried out as a part of one city's Suburb Project, prior to the nationwide study (data not shown). Some modifications were made to the questionnaire based on the comments received. The paper and internet questionnaire were identical and they were developed in parallel. On average it took 30 minutes to complete the questionnaire.

The final questionnaire included one hundred questions and it was divided into nine different sections as follows:

1) Respondent's information, seven items

2) Information about the place of residence, eight items

3) Information about the residence, 19 items

4) Hygiene, including drinking water, cleaning etc., 14 items

5) Physical and biological conditions, including ventilation, heating, dampness/moisture damage etc., 20 items

6) Chemical impurities, particles and fibres, 12 items

7) Safety, ten items

8) Welfare and health, seven items

9) Feedback, three items

### Sample size

The sample size was estimated in order to achieve 95% confidence level for the prevalence of common housing and health related factors, such as types of heating and ventilation systems, thermal conditions and perceived indoor air quality. At the end of 2007, there were approximately 2.73 million residences in Finland (Statistics Finland). Whilst the expected prevalence was not known with respect to all of the housing characteristics under review, using 50% for the estimated prevalence will result in the highest sample size, when required precision is set to ±3%. Therefore, the required sample size was approximately one thousand residences. In order to obtain the required data, a random sample of 3000 households was drawn from the Finnish Population Register Centre (FPRC) database.

### Ethical and data security issues

The study plan was ethically evaluated and an approval was obtained from the Ethical Committee of the National Institute for Health and Welfare. Participation in the study was voluntary. Privacy protection was in accordance with the Finnish Personal Data Act [16] as well as the requirements of the National Institute for Health and Welfare.

### Questionnaire data

A random sample of 3000 households was obtained from the FPRC. Persons aged 18 - 75 (one person per household-dwelling unit) were selected for the sample. They each received a mailed invitation to participate in the study with a paper questionnaire and instructions. The respondents could either complete and return the paper questionnaire by regular mail or complete the same questionnaire via the Internet. The electronic questionnaire was implemented through an Internet-based software service [17] and it was linked to the project's website, which was hosted on the Institute's web server [18]. The online survey was accessed via a secure connection.

### Response system

Depending on answers to specific questions regarding health and safety in the living environment, the respondents were given an option to receive individualised feedback. Feedback was available for 38 different items. For example, if someone responded that they had moisture or mould damage in their house, the feedback included general information on how such damage should be addressed. Technically, the response system was carried out with MySQL-database and PHP-scripting language. The response system analysed the questionnaire data taken from the Internet-based system and compiled individual feedback (sometimes for several issues) into a single document which was then sent to the respondent either by regular mail or by email as per the respondent's request.

### Questionnaire responses and data handling

The response rate was 25% after the first mailing. The questionnaire material was sent for a second time to those who had not yet responded. With the second mailing, a one-page form was also sent, which we asked to be returned if the respondent was still unwilling to complete the main questionnaire. The form requested the main reasons for not responding. After the second mailing of the questionnaire (cumulative response rate 43%), we sent a postcard to those who still had not responded encouraging them to answer the questionnaire via the Internet. The final response rate was 44%.

All data were entered into the Internet-based system either by the respondents or by the research personnel (with respect to the paper questionnaires). It was retrieved in CSV-format (comma-separated values), after which it was transferred to the SPSS-program and analysed with SPSS version 15.0. Descriptive statistics were drawn and 95% confidence intervals for point estimates were calculated using the formula:

p-1.96*√p*(1-p)/n) ≤ π ≥ p-1.96*√p*(1+p)/n), where p is the percentage value of the sample and n is the size of the sample.

### Spatial information

**Figure 1:**
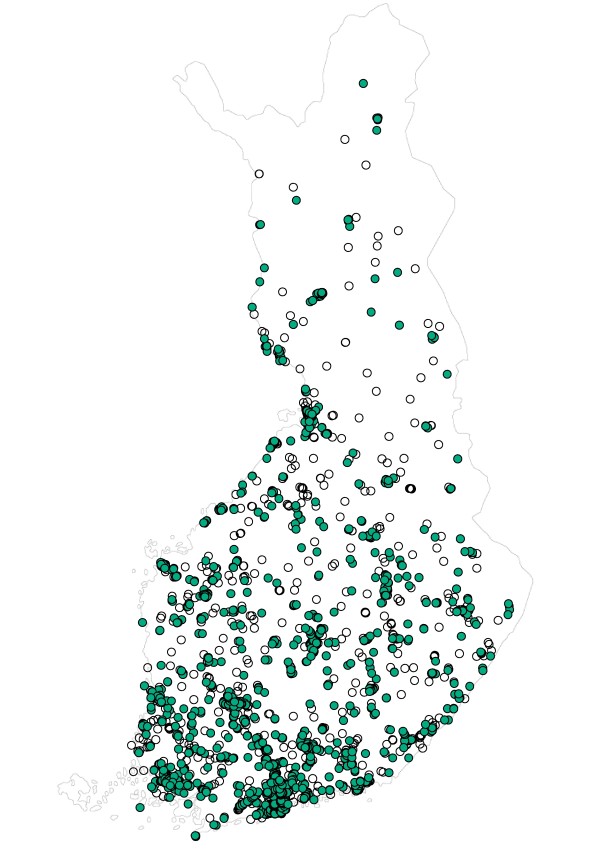
**Respondents**. The random sample (white dots) and the respondents (green dots) are illustrated on the map.

We also included spatial information in the database: Exact locations (coordinates) of each respondent's home, as well as respondent's socioeconomic information and general information about the residential buildings (e.g. year of construction, size of the residence) were obtained from the FPRC. Data from the FPRC and the questionnaire were then merged (see Figure 1 for an example). The merged data allowed responses to be mapped geographically and to be used in parallel with other mapped information (e.g. road/traffic maps, radon maps). Results from the spatial analyses will be reported in detail at a later date.

## Results

### Respondents

Figure 1 shows the random sample and the respondents mapped to geographical location. Altogether, we received 1312 responses, with 80 responding via the Internet. As a result of the low number of Internet responses, the results have been split by type of response mechanism only in this section. Internet respondents were younger on average than those who returned the paper questionnaire and a larger percentage were men (Table 1).

**Table 1:** Background information for respondents

	FPRC* sample (N = 3000)	All respondents (N = 1312)	Paper answers (N = 1232)	Internet answers (N = 80)
Age				

*Years*	46.5	48.8	49.3	40.8

Gender (N, %)				

*Female*	51.8	57.9	58.6	46.3

*Male*	48.2	42.1	41.4	53.8

Marital status (N, %)				

*Single*	35.6	15.2	14.5	26.3

*Married*	50.3	55.1	55.8	45.0

*Common-law marriage*	10.5	16.8	16.5	22.5

*Other*	4.6	12.8	13.3	6.3

Heating				

*District heating*	41.1	40.7	40.8	38.8

*Electricity*	26.2	28.7	28.7	30.0

*Wood*	10.8	17.1	17.5	11.3

*Oil heating*	19.9	13.9	13.8	15.0

*Geothermal heat*	0.4	0.8	0.8	0.0

Mechanical ventilation	39.5	37.8	37.3	46.3

* Finnish Population Register Centre

The overall response rate was 44%. Table 1 shows that compared to random sample as a whole, the respondents were older on average and a larger percentage were women. Some 55% of the respondents were married (50% in the random sample). However, concerning housing characteristics (e.g. heating, ventilation), the respondents did not differ significantly from the random sample.

### Background information for respondents and their place of residence

**Figure 2:**
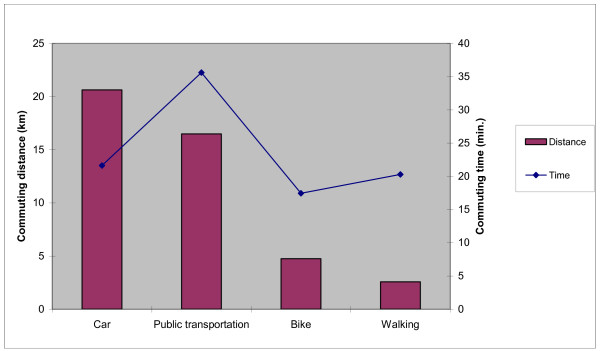
**Commuting time and distance**. The average times used for commuting.

A majority of the respondents were living in suburban areas, as shown in Table 2. The respondents were mostly satisfied or quite satisfied with the possibilities/public services in their local areas; public transportation was the most common cause of dissatisfaction (data not shown). Most respondents used a car to commute to work/school (41%), followed by cycling (15%), walking (14%) and finally using public transportation (11%). Figure 2 shows the average commute times.

**Table 2:** Background information about the respondents and their residences

	Respondents		Confidence interval 95%	
	**N**		**Lower Bound (%)**	**Upper Bound (%)**

Location of the residence				

*City centre*	181	14.0%	12.1	15.9

*Suburb*	530	41.0%	38.3	43.7

*Fringe area of the city*	154	11.9%	10.1	13.7

*Densely populated area*	203	15.7%	13.7	17.7

*Sparsely populated area, countryside*	225	17.4%	15.3	19.5

Years in current residence (mean)	1284	13.7	13.0	14.3

Owner-occupied	990	76.0%	73.7	78.3

Occupants per unit (mean)	1312	2.3		

Residence perceived large enough	1119	86.0%	84.1	87.9

Planning to move within a year	221	17.4%	15.3	19.5

Building characteristics				

*Flat roof*	218	16.9%	14.9	18.9

*Basement*	573	43.7%	41.0	46.4

*Energy efficient windows (triple glass or better)*	902	69.3%	66.8	71.8

Most common types of renovations in the past 12 months				

*Roofing*	61	4.6%	3.5	5.7

*Heating system*	61	4.6%	3.5	5.7

*Plumbing/sewage system*	56	4.3%	3.2	5.4

Pets				

*Dog or cat*	433	34.1%	31.5	36.7

Satisfied or quite satisfied with the indoor air quality	1169	90.1%	88.5	91.7

Ventilation				

*Mechanical supply and exhaust*	334	26.2%	23.8	28.6

*Mechanical exhaust*	325	25.5%	23.1	27.9

*Natural*	311	24.4%	22.0	26.8

Primary source of heating				

*District heating*	534	40.7%	38.0	43.4

*Electric heating*	377	28.7%	26.2	31.2

*Oil heating*	182	13.9%	12.0	15.8

**Figure 3:**
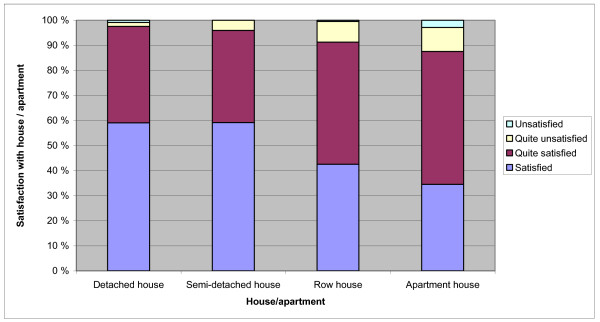
**Satisfaction with current house versus type of house**. The proportion of respondents satisfied with their residence in different types of residential buildings.

Figure 3 shows the distribution of satisfaction with the housing by type. Over 91% of the respondents reported being satisfied or quite satisfied with their residence, but the most satisfied were those living in detached houses.

Respondents had been living on average for 13.7 years in their current home, as shown in Table 2. A majority of the residences were owner-occupied. The average size of the household per dwelling unit was 2.3 persons, which is slightly higher than expected based on the 2006 data from Statistics Finland (average 2.1 persons). About 86% of the respondents thought their home was large enough. However, about a fifth of respondents were planning to move within the next 12 months, and the main reason for moving was the size of the residence. The most common types of renovation carried out in the previous 12 months related to the roof, heating system, and/or plumbing/sewage system.

As presented in Table 2, almost 90% of the respondents were satisfied or quite satisfied with the indoor air quality in their home. Most of the respondents reported having either mechanical ventilation or mechanical exhaust; and 24% had natural ventilation. Some 11% reported that they had no ventilation at all and an equal percentage did not know what kind of ventilation they had. The most common primary heating system was district heating (for an explanation of district heating see abbreviations), while the second was electric heating, and the third was oil heating.

### Hygiene equipment and practices

Table 3 shows that over 85% of the respondents received their drinking water from municipal water supplies. One in ten respondents had noticed some unusual taste, smell, sediment, or colour in their drinking water. Almost a third of the respondents had reported water supply disruptions within the last 12 months, most commonly caused by either repair work or faults in the water system.

**Table 3:** Hygiene equipment and practices

	Respondents		Confidence interval 95%	
	
	N	%	Lower Bound (%)	Upper Bound (%)
Drinking water supply				

*Municipal water supplies*	1110	85.6	83.7	87.5

*Own well*	125	9.7	8.1	11.3

Have noticed unusual taste or something else in the drinking water	140	10.8	9.1	12.5

Water supply disruptions, due to				

*Repair work*	267	22.9	20.5	25.3

*Faults in the water system*	68	6.1	4.7	7.5

Cleaning tasks performed weekly or more often				

*Taking trash out*	1259	99.6	99.3	99.9

*Laundry*	1225	96.7	95.7	97.7

*Vacuuming*	1167	91.3	89.8	92.8

Seen signs of				

*Rodents indoors*	98	7.5	6.1	8.9

*Insects (herculean ant etc.) indoors*	137	10.4	8.7	12.1

A majority of the respondents did laundry, vacuuming and household waste removal at least weekly. In regard to general hygiene, 8% of respondents had seen signs of rodents indoors and 20% had seen signs of rodents in their yard.

### Physical and biological conditions

A total of 57% of the respondents had fresh air intake vents in their bedroom and over 70% ventilated their residence daily by opening the windows as detailed in Table 4. Only a small proportion of respondents had a humidifier or an air purifier in their residence. In regard to combustion, 34% of the respondents had a fireplace, 23% a wood stove and 19% a wood-burning sauna stove in their home, with only 3% having a gas stove.

**Table 4:** Physical and biological conditions

	Respondents		Confidence interval 95%	
	
	N	%	Lower Bound (%)	Upper Bound (%)
Fresh air intake vents in the bedroom	735	57.3	54.6	60.0

Ventilation enhanced daily by opening the windows	925	73.1	70.7	75.5

Humidifier	67	5.1	3.8	6.4

Air purifier	98	7.5	6.0	9.0

Combustion				

*Fireplace*	451	34.4	31.7	37.1

*Wood stove*	300	22.9	20.6	25.2

*Wooden sauna stove*	244	18.6	16.4	20.8

*Gas stove*	44	3.4	2.4	4.4

Severe water damage in the past 12 months	33	2.6	1.7	3.5

Damage repaired by				

*Removing the damaged materials*	99	7.5	3.6	11.4

*Drying the structures*	94	7.2	3.3	11.1

*No actions have been taken*	26	2.0	-0.1	4.1

Current moisture or mould damage	70	5.3	4.1	6.5

Size of the damage is large		0.1 - 0.5		

Main cause of the damage				

*Inside sources (plumbing leak etc.)*	30	2.3	-0.7	5.3

*Outside sources (roof leaks etc.)*	23	1.8	-0.9	4.5

*Cause unknown*	29	2.2	-0.7	5.1

Lighting deficiencies	524	39.9	37.2	42.6

Daily noise caused by road traffic	276	22.4	20.1	24.7

Occasional/seasonal noise caused by yard work	330	28.6	26.0	31.2

Eleven per cent reported that they had some serious water damage in their residence and 3% of those had occurred within the last 12 months. Usually the damage had been repaired by removing the damaged materials or by drying the structures, although 2% reported that no actions had been taken.

At the time of the survey, moisture or mould damage was reported in 5% of the residences, a majority being described as local damage in the bathrooms. The main cause of moisture or mould damage was water from inside sources (plumbing leaks, etc.), while an almost equally common cause was water from outside sources (rain water, roof leaks, etc.).

A total of 39% of respondents reported some deficiencies in the lighting of their neighbourhood. The main source of daily noise was caused by road traffic, whereas the most common occasional/seasonal noise source was caused by yard work.

### Chemical impurities, particles and fibres

About 2% of the respondents reported smoking in their home on a daily basis as presented in Table 5. Over 28% were using insecticides and 17% were using herbicides in their household, but the usage was described as occasional. The most unpleasant outdoor odours that were reported related to traffic, farming, smoking, industry, and smoke; whereas the most common indoor odours related to food, sewers, stuffy air, and tobacco smoke. About 30 - 40% of the respondents had not noticed any odours in their home or in the neighbourhood.

**Table 5:** Chemical impurities, particles and fibres

	Respondents		Confidence interval 95%	
	
	N	%	Lower Bound (%)	Upper Bound (%)
Smoking inside the residence daily				

*Respondent him/herself*	32	2.5	1.6	3.4

*Someone else*	27	2.2	1.4	3.0

Using insecticides	336	28.5	25.9	31.1

Using herbicides	161	16.7	14.3	19.1

Unpleasant odours outside				

*Traffic*	136	10.4	8.6	12.2

*Farming*	128	9.8	8.1	11.5

*Smoking*	119	9.1	7.4	10.8

*Industry*	116	8.8	7.2	10.4

*Smoke*	101	7.7	6.2	9.2

*No odours outside*	395	30.1	27.5	32.7

Unpleasant odours inside				

*Food*	112	8.5	6.9	10.1

*Sewer*	75	5.7	4.4	7.0

*Stuffy air*	73	5.6	4.3	6.9

*Tobacco smoke*	65	5.0	3.7	6.3

*No odours inside*	587	44.7	41.8	47.6

Asbestos				

*In living areas*	10	0.8	0.3	1.3

*Outside living areas*	52	4.1	3.0	5.2

*Unaware of the asbestos*	362	28.8	26.3	31.3

Radon				

*Elevated radon levels*	9	0.7	0.2	1.2

*Unaware of the radon*	830	65.8	63.2	68.4

Less than 1% of the respondents reported that there was asbestos in their living areas and 4% reported asbestos outside the living areas (for example in the basements of the block of flats). A few respondents reported elevated radon levels in their residence, but over 65% were unaware of the radon status for their place of residence.

### Safety, welfare and health

Concerning the safety issues, Table 6 shows that over 96% of the respondents felt safe or quite safe in their neighbourhood. Fewer than 3% had felt personally threatened and fewer than 4% reported that their home had been broken into within the last 12 months.

**Table 6:** Safety, welfare and health

	Respondents		Confidence interval 95%	
	
	N	%	Lower Bound (%)	Upper Bound (%)
Feeling safe/quite safe in the neighbourhood	1255	96.9	96.0	97.8

Within the last 12 months				

*Had felt personally threatened*	33	2.6	1.7	3.5

*Residence had been broken into*	48	3.7	2.7	4.7

Safety equipment				

*Fire alarm*	1273	97.0	96.1	97.9

*Fire extinguisher*	739	56.3	53.6	59.0

*First aid kit*	683	52.1	49.4	54.8

Accidents in the neighbourhood				

*Falls/slips*	152	11.6	9.9	13.3

*Fires*	59	4.5	3.4	5.6

Neighbourhood is				

*Accessible*	333	26.5	24.1	28.9

*Not accessible*	245	19.5	17.3	21.7

Perceived health good/quite good	972	75.7	73.4	78.0

Daily symptoms				

*Upper respiratory symptoms*	129	11.1	9.4	12.8

*Muscular pain*	110	9.7	8.1	11.3

*Skin symptoms*	104	9.3	7.7	10.9

*Eye symptoms*	105	9.1	7.5	10.7

*General symptoms*	101	8.8	7.2	10.4

*Sleeping problems*	85	7.5	6.0	9.0

*Lower respiratory symptoms*	75	6.7	5.3	8.1

Doctor diagnosed asthma	98	7.7	6.2	9.2

Allergies				

*Hay fever*	212	17.8	15.6	20.0

*Pet allergy*	125	10.9	9.1	12.7

Within the last 12 months				

*At least one episode of respiratory infections*	276	22.4	20.1	24.7

*Has visited a doctor because of respiratory symptoms*	266	21.8	19.5	24.1

*Had missed work/school days due to respiratory infections*	168	15.0	12.9	17.1

Almost all the respondents had a fire alarm in their home and more than half had also a fire extinguisher and a first aid kit. The most common accidents that occurred in the respondents' home or neighbourhood were falls/slips and fires. A quarter of respondents thought that their neighbourhood was accessible; whereas some 20% reported the neighbourhood was not accessible.

Concerning health issues, about 75% of the respondents perceived their health to be good or quite good. The most common daily symptoms were respiratory symptoms (upper respiratory tract) and/or arthralgia; the second most common daily symptoms were general symptoms, eye and skin symptoms and muscular pain. Seven per cent had regular sleeping problems and 6% reported daily lower respiratory tract symptoms.

Seven per cent of the respondents had doctor-diagnosed asthma. The most common allergies were hay fever and pet allergy. Within the last 12 months, 22% had at least one episode of respiratory infections, 22% had visited a doctor and 15% had missed work/school days due to respiratory infections.

### Information needs

The questionnaire was divided into nine different sections as described in the paragraph 'Questionnaire development'. At the end of Sections 2-7, there was a question about information needs concerning the topics covered in the section. In addition, in Section 9 (Feedback) there was also a question related to the need for information/counselling services.

Table 7 shows that regarding the place of residence (Section 2), altogether 25% of the respondents felt that they would need more information about factors relating to health and safety issues in their local residential areas. In regard to health and safety issues in the dwelling itself (Section 3), some 27% of the respondents reported needing more information.

**Table 7:** Information needs, including information/counselling services

Question	Respondents	
	
	N	%
Do you need information concerning safety/healthiness issues of your living area?		

*No*	936	71.3

*Yes*	322	24.5

Do you need information concerning safety and healthiness issues of your residence?		

*No*	917	69.9

*Yes*	350	26.7

What information do you need concerning the following housing and health issues (hygiene part)?		

*Drinking water quality*	341	26.0

*Rodents and other pests*	143	10.9

*Waste water treatment*	124	9.5

What information do you need concerning the following housing and health issues (physical and biological issues)?		

*Ventilation*	316	24.1

*Dampness/mould*	253	19.3

*House maintenance*	224	17.1

*Heating system*	135	10.3

What information do you need concerning the following housing and health issues (chemical impurity-, particle- and fibre -issues)?		

*Radon*	303	23.1

*Indoor air pollutants*	301	22.9

*Asbestos*	137	10.4

What information do you need concerning the following housing and health issues (safety issues)?		

*Safety of the neighbourhood*	170	13.0

*Security systems*	167	12.7

*Safety of the walking trails*	111	8.5

Would you like to have individualised feedback based on your answers?		

*No*	879	67.0

*Yes, by mail*	286	21.8

*Yes, by email*	147	11.2

What kind of information/counselling services would you need related to housing and health issues?		

*Municipal services*	283	21.6

*Consultant services*	34	2.6

*Complimentary counselling services*	395	30.1

*Services of housing, construction and maintenance companies*	182	13.9

*Internet-based services*	234	17.8

*No need for such services*	546	41.6

*Something else*	14	1.1

In relation to hygiene issues (Section 4), respondents most commonly reported a need for information about drinking water quality, waste water treatment, and rodents and other pests. Concerning physical and biological conditions in Section 5, the most commonly reported need was for information about ventilation, dampness/mould damage, and building maintenance issues.

In Section 6 (Chemical impurities, particles and fibres), respondents most commonly needed information about radon and indoor air pollutants, as well as asbestos. In Section 7 (Safety), respondents most commonly reported needing information about safety in the neighbourhood, security systems, and also about the safety of pedestrian paths.

Table 7 shows that in regard to information/counselling services (part of Section 9); most respondents reported needing information about complimentary counselling services and municipal services.

### Response system analysis

**Figure 4:**
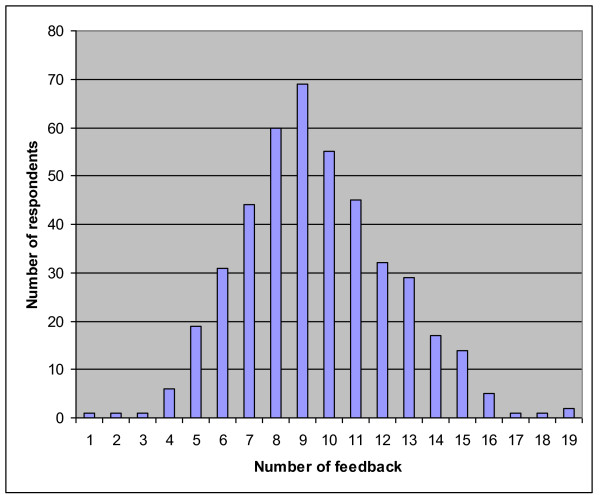
**Distribution of feedback**. Distribution of the number of feedback received by respondents.

About a third of respondents (33%) wanted feedback based on their responses. Altogether, it was possible to get feedback on 38 question responses. As seen on Figure 4, the maximum number of feedback per respondent was 19 feedbacks, and on average, feedback was given to 10 responses. Feedback was sent both by email

(34%) and by regular mail (66%). Those respondents who returned the paper questionnaire preferred to have the feedback by regular mail (71%) rather than by email (29%). On the contrary, a majority of the respondents who answered through the Internet were also more likely to want their feedback through email (80%). As shown in Table 8 the most prevalent feedback requests (over 50%) concerned issues such as ventilation, foul taste or odour in the drinking water, radon, cleaning, pollen sources, and thermal conditions in the residence.

**Table 8:** Ten most prevalent feedback topics related to questionnaire responses

Rank	Feedback concerning
1	Ventilation

2	Foul taste or odour in the drinking water

3	Radon

4	Cleaning

5	Pollen sources

6	Temperature conditions in the residence

7	Cookers/fireplaces

8	Security systems (inc. fire and burglar alarms)

9	Sources that can cause disturbance and/or pollution (for example roads and factories)

10	Asbestos

## Discussion

In this study, the total response rate was 44%, which is relatively good when compared with other self-administered occupant questionnaire studies that are related to health and safety issues [19-21]. General information about residences showed that the data collection and response system was able to collect information that was representative of the housing stock. Based on the 95% confidence intervals, the estimates drawn based on this sample were 0.7% to 7.8% accurate.

Even though only a small proportion of answers came via the Internet, it is still considered a valuable tool to be used in questionnaire studies in the future. Its advantages include better controllability of the data and cost savings in mailing, data recording and filing tasks. Internet-based systems could potentially also be used in longitudinal studies in the future.

One reason for the low Internet response rate could be the length of the questionnaire. It might be easier for respondents to complete the paper version over several shorter periods than to complete it all at once (average response time about 30 minutes) as was necessary with the online survey. If it was possible to send a link directly to the respondents' email addresses, it could have improved the Internet response rate. Also those who responded via the Internet were typically younger, single, more educated, and a larger proportion of them were male. It could be speculated that the Internet questionnaire may have improved the response rate for such sections of the population, whereas the 'typical' respondents have been older, married women. However, Internet-based questionnaires might currently fit better for certain target populations than the general public.

We were also able to obtain spatial data (exact location of the respondent's place of residence) in the study. It can be used to explore additional information about housing and health issues, for example in terms of visualisation and mapping (see Figure 1).

The place of residence is an important factor that could be connected to housing health and safety both directly and indirectly. For example, Wilson et al. [22] have reported that a place of residence (neighbourhood) has limited importance for determining health, but perceptions of neighbourhood environments (i.e., dislikes and to a lesser extent likes) are important for some health outcomes.

Place of residence is naturally related to socioeconomic status, but it may also determine the availability of municipal water and sewage systems, services and transportation. Home location as such can have many implications for health and safety. For example, a home location that enables the occupants to commute by bike or to walk may promote their health more than many other 'healthy' housing parameters. A location that enables the occupants to use public transportation rather than a car may be environmentally friendly. However, public transportation also commonly results in dissatisfaction that may be reflected in users' responses about their living environments.

Of the respondents, 32% were living in blocks of flats, 44% were living in detached houses, 14% in row houses and 4% in semi-detached houses. According to Statistics Finland in 2005, 44% of the households were living in blocks of flats, 40% in detached houses, and 14% in row houses. Hence, more of this study's respondents were living in detached houses and fewer in blocks of flats compared to the statistical population data from 2005.

In addition to the type of residence, other important general housing health and safety aspects may include the size of residence and/or the occupant's perception of the size, as well as time spent living in the residence, plans to move, and renovation status. For example, occupancy per square meter is an objective way to assess crowdedness. We also asked whether the occupants perceived their home as large enough, although that element is more subjective. Time of living in a residence could be attributed to exposure time for certain environmental conditions, although they are subject to change. In addition to general life changes (relocating because of work, family situation, etc.), plans to move may be related to general dissatisfaction or observed defects in housing conditions. Completed or planned renovations may also be related to occupant satisfaction and well-being.

Drinking water quality is, in general, considered to be high in Finland. Over 85% of residences are covered by municipal water and sewage services. However, about 10% of respondents had noticed an unusual smell, taste, sediment or colour in their drinking water. Moreover, almost one-third reported water supply disruptions, mainly due to repairs.

Regular cleaning is considered important for housing health and quality, even though there is not much information about typical cleaning practices and their frequency. House dust contains microbes such as bacteria and fungi [23-25], as well as other impurities. Pets can increase concentrations of various pollutants in the home and they may cause allergic and asthmatic symptoms [26]. In this study a majority of respondents reported performing basic cleaning tasks on a regular basis, such as taking the trash out and vacuuming. The Internet respondents did some of the cleaning tasks less frequently than those who returned the paper questionnaire, which may be related to factors such as gender, age, and socioeconomic status (data not shown).

According to new building regulations, residential buildings must have a sufficient ventilation/air exchange rate [27]. Modern building envelopes are becoming more airtight, so it is difficult to obtain a sufficient level of ventilation without a properly functioning mechanical system. Considering that fewer than 27% (Table 2) of the respondents reported having mechanical support and an exhaust ventilation system in their residence, only a part of the current housing stock would fulfil the criteria for sufficient ventilation. It could partly explain why a large part of the respondents enhance ventilation by opening windows, which in turn may result in energy loss, particularly during winter.

In Finland, electric heating and oil heating are still common although renewable energy sources are generally supported [28]. While electric heating can also be renewable, in Finland only 15% of electricity consumption was provided for by hydropower and only 0.3% by wind power in 2009 [29]. The proportion of renewable sources is expected to rise in the future, which may also have an effect on exposures to pollution originating from indoor and outdoor sources. Therefore it is important to also follow up trends in heating systems and usage (both primary and complementary) from an environmental health perspective.

Our results for the occurrence of moisture and mould damage (5%) are a little lower than reported in other studies. For example, Kilpeläinen et al. found out that visible mould or damp stains or water damage was reported by 15% of respondents [30]; whereas in other studies the occurrence of moisture damage in residential buildings has previously been approximately 20-40% [4,31]. It is possible that the increase in the general awareness of the association between moisture and mould damage and health has led to improved maintenance and better repair of buildings in recent years. Interestingly, 19% of respondents reported specifically needing more information about moisture and mould related issues, so it still seems to be a matter of concern.

The health effects of noise have been widely studied, mainly in the form of annoyance and sleep disturbance, although cardiovascular effects have also been reported [32]. Lighting is considered to be an important factor related to the feeling of safety, and the lack of light during the winter has also been associated with depression [33]. From 20-30% of the respondents had experienced some disturbance because of noise or lack of light.

People are sensitive to unpleasant odours, which may cause disturbance, irritation symptoms, general symptoms and nausea. Odours have also been suggested as an important cause of worsening of asthma [34]. In this study, the most commonly reported unpleasant indoor odours were related to food, sewer gas, stuffy air, and tobacco smoke; they could also be related to insufficient or unbalanced ventilation.

Asbestos has been widely used in many building materials, especially during the 1960s and 1970s. Exposure to asbestos can cause lung cancer and asbestosis [35] for example. Less than 1% of respondents reported that there was asbestos in their living areas. However, 28% of the respondents did not know whether there was asbestos, while 10% reported needing more information about asbestos, so it may still be a matter of concern.

In Finland, indoor radon concentrations are among the highest in the world. The reasons for high concentrations are, among others, elevated uranium concentration in the ground and the cold climate [36]. Radon increases the risk for lung cancer [37]. In this study it was considered noticeable that over half of the respondents were unaware of the radon situation in their place of residence, and 23% reported needing more information about radon.

In regard to safety issues, a majority of the respondents felt safe or quite safe in their neighbourhood and fewer than 3% had felt personally threatened within the last year. According to another Finnish study from 2006, a total of 68% of the respondents had felt safe while walking in the centre of their hometown late on Friday and Saturday nights and about 14% had felt unsafe in their neighbourhood during weekend nights [38].

Approximately a quarter of the respondents felt that they would need more information about factors relating to health and safety issues in their local residential areas and also for their residence. Of the more specific housing and health related factors, the most frequently reported information needs related to drinking water quality (26%), ventilation (24%), indoor air pollutants and radon (23%) and dampness/mould (19%). In regard to the types of information/counselling services, respondents mostly reported needing more information about complimentary counselling services, with consulting services considered less useful.

The response system was an important part of this study. It gave us the means to provide important information about housing health and safety issues for the respondents. We also wanted to raise respondent's awareness and encourage them to look for more information concerning relevant housing issues. In addition, providing the incentive of feedback was decided with the aim of increasing the response rate. Research ethics are quite strict in Finland, which prevents us from offering monetary incentives, gift card, presents, etc. We asked the respondents to spend a significant amount of time in answering the questionnaires. Therefore it feels 'appropriate' to be able to offer something for them in return. This approach may therefore offer multiple benefits.

The feedback provided was developed as a combined effort linking the general guidance available on housing health and safety issues in Finland. The sources quoted included building codes [27], a housing and health guideline and guidebook [13,14], the Finnish Classification of Indoor Climate [39], and guidance material developed by The Radiation and Nuclear Safety Authority, the Finnish Allergy and Asthma Federation, the Pulmonary Association HELI, and other non-profit organisations.

Given that a third of the respondents opted to receive feedback for specific question items, it raises the question of whether the feedback option may have motivated occupants residing in 'bad' housing in particular to participate in the survey. We tested this briefly by comparing the results of those asking for feedback with those not wanting feedback. Although the general characteristics of these two groups were similar (no observed differences in age, gender, socioeconomic status, general building characteristics, etc.), there were statistically significant differences in some of the housing variables tested: feedback was requested more frequently among those who reported water damage, who had mechanical ventilation systems, and/or those who did not know about the radon situation in their place of residence. These results will be reported in detail elsewhere.

The response system was developed so as to automate the analysis and compiling work thereby reducing labour intensiveness and allowing the study personnel to send the feedback in a timely fashion. Unfortunately, we do not know for sure whether the respondents acted on the feedback they received; a follow-up study relating to this issue has been planned.

## Conclusions

We have collected a largely representative housing database, which includes comprehensive information on housing quality and housing health and safety in Finland. This can be used to explore housing issues in relation to health and well-being. It appears that collecting questionnaire data while including the possibility of receiving feedback encourages some people to respond and request more information about their housing conditions. However, data collection relying only on the Internet does not appear to result in a good response rate in a general population sample at this point in time.

## List of Abbreviations

CSV: comma-separated values; District heating: District heating is a system for distributing heat generated in a centralized location for residential and commercial heating requirements. It is produced in Combined Heat and Power (CHP) plants or heating plants. Customers receive heat from the hot water circulating in the heat distribution system and the heat is used in houses for the heating of rooms and service water, as well as for ventilation. District heating is the most common form of heating in Finland; FPRC: Finnish Population Register Centre; KTL: National Public Health Institute; MySQL-database: MySQL is a relational database management system. The MySQL-database runs on server which provides access to the database; PHP: PHP Hypertext Preprocessor is a scripting language which is used widely in web-based systems; SPSS: Statistical Package for the Social Sciences (SPSS is also name of a company); THL: National Institute for Health and Welfare

## Competing interests

The authors declare that they have no competing interests.

## Authors' contributions

MT participated in designing the questionnaire and the data collecting system, handling the questionnaires, performed part of the statistical analyses and wrote the first draft of the manuscript. AP participated in designing the questionnaire and the data collecting system, handling the questionnaires, performed spatial analyses, and participated in writing the manuscript. JV participated in designing the response system, sending feedback to the participants, and commenting the manuscript. AN participated in overseeing the study and in writing the manuscript. UHS participated in designing and co-ordinating the study and in writing the manuscript. All authors read and approved the final manuscript.
